# BET bromodomain protein inhibition is a therapeutic option for medulloblastoma

**DOI:** 10.18632/oncotarget.1534

**Published:** 2013-10-27

**Authors:** Anton Henssen, Theresa Thor, Andrea Odersky, Lukas Heukamp, Nicolai El-Hindy, Anneleen Beckers, Frank Speleman, Kristina Althoff, Simon Schäfers, Alexander Schramm, Ulrich Sure, Gudrun Fleischhack, Angelika Eggert, Johannes H. Schulte

**Affiliations:** ^1^ Department of Pediatric Oncology and Hematology, University Children's Hospital Essen, Essen, Germany; ^2^ German Cancer Consortium (DKTK), Germany; ^3^ Translational Neuro-Oncology, West German Cancer Center, University Hospital Essen, University Duisburg-Essen, Essen, Germany; ^4^ German Cancer Research Center (DKFZ), Heidelberg, Germany; ^5^ Centre for Medical Biotechnology, University Duisburg-Essen, Essen, Germany; ^6^ Institute of Pathology, University Hospital Cologne, Cologne, Germany; ^7^ Department of Neurosurgery, University of Essen, Essen, Germany; ^8^ Center of Medical Genetics Ghent (CMGG), Ghent University Hospital, Ghent, Belgium; ^9^ Department of Pediatric Oncology/Hematology, Charité-Universitätsmedizin Berlin, Germany

**Keywords:** BET bromodomains, BRD4, MYC, JQ1, pediatric brain tumors, targeted therapy

## Abstract

Medulloblastoma is the most common malignant brain tumor of childhood, and represents a significant clinical challenge in pediatric oncology, since overall survival currently remains under 70%. Patients with tumors overexpressing *MYC* or harboring a *MYC* oncogene amplification have an extremely poor prognosis. Pharmacologically inhibiting *MYC* expression may, thus, have clinical utility given its pathogenetic role in medulloblastoma. Recent studies using the selective small molecule BET inhibitor, JQ1, have identified BET bromodomain proteins, especially BRD4, as epigenetic regulatory factors for *MYC* and its targets. Targeting *MYC* expression by BET inhibition resulted in antitumoral effects in various cancers. Our aim here was to evaluate the efficacy of JQ1 against preclinical models for high-risk MYC-driven medulloblastoma. Treatment of medulloblastoma cell lines with JQ1 significantly reduced cell proliferation and preferentially induced apoptosis in cells expressing high levels of MYC. JQ1 treatment of medulloblastoma cell lines downregulated *MYC* expression and resulted in a transcriptional deregulation of MYC targets, and also significantly altered expression of genes involved in cell cycle progression and p53 signalling. JQ1 treatment prolonged the survival of mice harboring medulloblastoma xenografts and reduced the tumor burden in these mice. Our preclinical data provide evidence to pursue testing BET inhibitors, such as JQ1, as molecular targeted therapeutic options for patients with high-risk medulloblastomas overexpressing *MYC* or harboring *MYC* amplifications.

## INTRODUCTION

Tumors originating in the central nervous system (CNS) are the second most prevalent cancers and the leading cause of cancer-related mortality in childhood [1]. Medulloblastoma is the most common malignant brain tumor in children and still presents a significant challenge to treat, with an overall survival under 70% [2]. Four subgroups with distinct clinical, biological and genetic profiles are now recognized, namely, WNT, SHH, group 3 and group 4 [3]. WNT as well as Group 3 tumors express high MYC levels [3]. Group 3 is the most aggressive subgroup and typically expresses the highest *MYC* levels, with *MYC* amplifications occurring almost exclusively in this subgroup [4-6]. This most aggressive form of medulloblastoma portends a dismal prognosis, and generates a high proportion of aggressive, invasive and metastasizing tumors [4, 5, 7, 8]. Group 3 tumors are usually resistant to even multimodal treatment consisting of surgery, radiotherapy and chemotherapy. Thus, the integration of molecular targeted therapies into current treatment protocols and adjustment of conventional treatment is urgently needed to improve survival in patients with high-risk medulloblastoma without compromising long-term quality of life after treatment. As high-level MYC expression may drive the most aggressive characteristic of medulloblastomas, targeted inhibition of *MYC* should have clinical utility.

Posttranslational histone modifications are crucial for the modulation of chromatin structure and regulation of transcription [9]. Deregulation of these epigenetic modifications is common among cancer cells, and can lead to overexpression of oncogenes [10]. Bromodomains recognize acetylated lysines in the N-terminal regions of histones and, thus, function as chromatin readers [11] within the read-write-erase concept for the transfer of epigenetic information. Proteins containing reader domains recruit enzymes that add or remove posttranslational modifications, the writers and erasers, respectively, to the chromatin at areas of lysine modification. The BET protein family contain tandem bromodomains and an extraterminal or ET domain [12]. Human BET family members include BRD2, BRD3, BRD4 and BRDT [12]. BRD2, BRD3 and BRD4 are ubiquitously expressed, whereas, BRDT is localized primarily to the testis [13]. The BRD2 and BRD3 proteins have been shown to regulate the transcription of growth-promoting genes such as *CCND1*, suggesting that they have reinforcing roles on controlling proliferative expansion [14]. BRD4 is a well-established regulator of the positive transcription elongation factor b (P-TEFb), a complex consisting of cyclin-dependent kinase 9 (CDK9) and cyclin T, among other polypeptides [15]. Through its interaction with BRD4, P-TEFb is recruited to promoters to phosphorylate the carboxy-terminal domain of the large subunit of RNA polymerase II. Functional studies have suggested that BRD4 plays an important role in regulating growth-associated genes at the M/G1 boundary by retaining P-TEFb at the promoters of key regulatory genes throughout mitosis [16, 17]. Furthermore, P-TEFb is recruited to *MYC* and MYC target gene promotors as an important step for MYC-dependent stimulation of response genes. One such response gene is *CAD*, which encodes a protein containing the first three enzyme activities of pyrimidine nucleotide biosynthesis, carbamyl phosphate synthetase II, aspartate transcarbamylase and dihydro-orotase [18, 19]. In summary, BET proteins play an important role in controlling proliferation and cell cycle progression, making them interesting targets for cancer therapy.

Several reports in the last two years have demonstrated antitumorigenic activities of the small molecule BET bromodomain inhibitor, JQ1, in preclinical models of hematological malignancies and neuroblastoma [20-22]. JQ1 binds competitively to bromodomains to displace them from acetylated histones, and shows highest potency and specificity towards BRD4, with weaker binding to BRD2, BRD3 and BRDT [23]. Intriguingly, BRD4 was shown to bind to histones in the *MYC* promoter region itself and play a critical role in *MYC* expression in human cancer cells such that inhibition of BET with JQ1 resulted in a remarkable diminution of *MYC* expression, decreased BRD4 binding to the *MYC* promotor and associated cell death [20, 21]. Inhibition of the BRD4 protein by JQ1, thus, proved to have effective antitumoral properties, suggesting that targeting *MYC* expression is feasible in selected cancers [20, 21].

With the aim of exploring molecular targeted therapeutic options for high-risk medulloblastomas, we analyzed whether inhibiting BET bromodomain proteins, and thereby targeting *MYC* and its target genes, could be effective against preclinical models of medulloblastoma. We hypothesized that especially high-risk medulloblastomas, which overexpress *MYC*, could benefit from BET inhibition. To provide proof-of-principle that BET inhibition is therapeutically useful against medulloblastoma, we assessed the efficacy of JQ1 against medulloblastoma cells grown in cell culture models and as xenografts in nude mice.

## RESULTS

### BRD4 is expressed in primary medulloblastomas and human medulloblastoma cell lines

**Figure 1:**
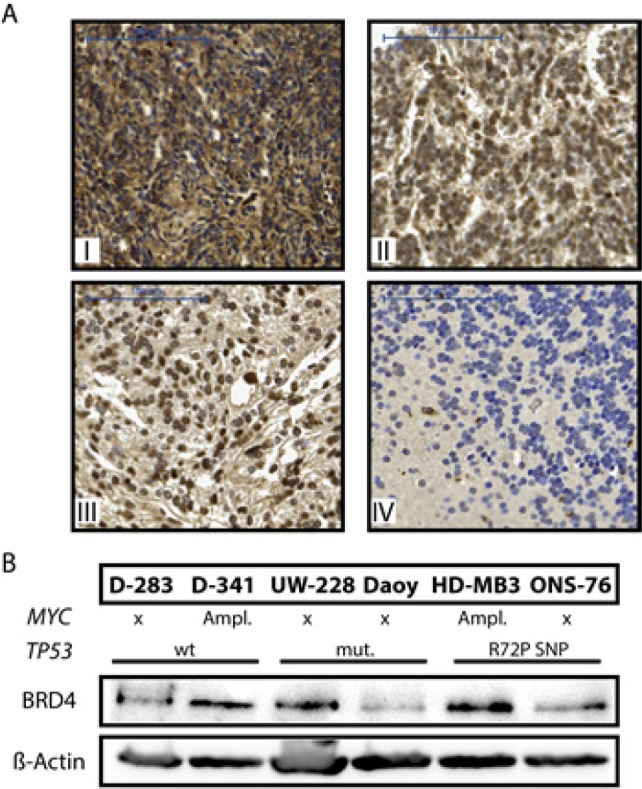
BRD4 is expressed in primary medulloblastomas and medulloblastoma cell lines A. BRD4 expression was immunohistochemically analyzed in primary medulloblastomas from 115 pediatric and 2 adult patients and in 14 samples of normal cerebellum as a control tissue. Four exemplary pictures of BRD4 staining in medulloblastomas and normal human cerebellum are shown: I-II from pediatric medulloblastomas, III from an adult medulloblastoma and IV from cerebellum. B. BRD4 protein expression was analyzed in western blots of whole-cell lysates from a panel of human medulloblastoma cell lines. HD-MB3 and D-341 harbor *MYC* amplifications and UW-228 as well as Daoy harbor *TP53* mutations. β-actin was used as a loading control.

BRD4 was previously shown to be the most sensitive of all BET proteins to JQ1 treatment in *in vitro* pharmacological assays. We therefore assessed BRD4 expression in primary medulloblastomas and normal cerebellar tissue, as a control. BRD4 was immunohistochemically detected in samples from 115 primary medulloblastomas from pediatric patients, 14 cerebellum samples previously arrayed into a tissue microarray and 2 samples from primary medulloblastomas from adult patients. High-level BRD4 expression were detected in 99 of 115 pediatric primary medulloblastomas (75%) and in both adult medulloblastoma samples (Fig. 1A, I-III). BRD4 was only marginally (22%) expressed in normal cerebellar tissue (Fig. 1A, IV and supplementary Fig. 1). We also evaluated BRD4 expression in a panel of medulloblastoma cell lines that included HD-MB3, ONS-76, UW-228, Daoy, D-341 and D-283. All cell lines strongly expressed BRD4 (Fig.1 B), except for Daoy, which expressed lower BRD4 levels. Robinson, et al. previously published global mRNA expression profiles from 76 primary medulloblastomas and 9 normal cerebellar samples performed on Affymetrix chips [24]. We re-analyzed these data, and detected significantly higher *BRD3* expression levels in medulloblastomas compared to normal human cerebellum (P <0.01). *BRD2* and *BRD4* were both expressed in all primary medulloblastomas in this medulloblastoma cohort, however, expression was not significantly higher than in cerebellar tissue. Taken together, *BRD2, BRD3* and *BRD4* are expressed in primary medulloblastomas. All medulloblastoma subgroups strongly expressed BRD4, with the highest expression detected in the shh subgroup (see supplementary Fig. 2). While expression of only *BRD3* was elevated in tumors from the cohort analyzed, BRD4 expression was significantly higher in tissue samples from a separate medulloblasoma cohort as well as in cell lines derived from human medulloblastomas, indicating that mRNA expression may not be the best measure of protein activity and targetability in this case. The one prerequisite for our study assessing JQ1 efficacy against medulloblastoma, that its primary target be expressed, was fulfilled not only in our preclinical models but also in primary medulloblastoma samples.

### JQ1 reduces cell viability and proliferation and induces apoptosis and senescence in human medulloblastoma cell lines

**Figure 2:**
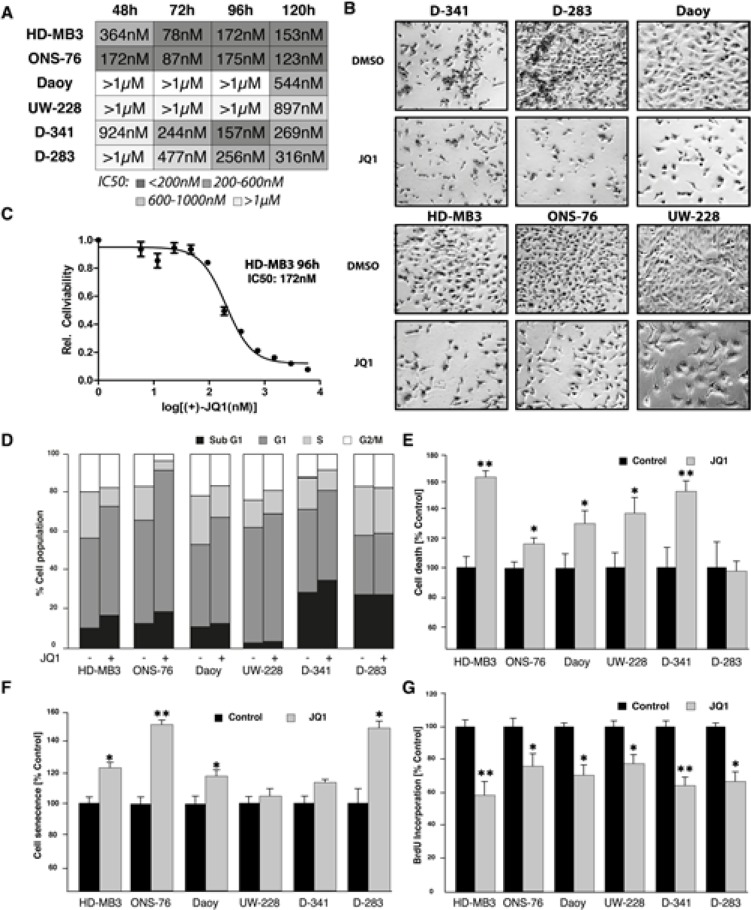
JQ1 treatment reduces viability of medulloblastoma cells A. Absolute IC_50_ values of all six cell lines at different time points after treatment wich JQ1. B. Images taken from cells treated with JQ1 at IC_50_ concentration or with DMSO (control). C. Exemplary dose-response curve for HD-MB3 cells treated with JQ1 for 96h is shown. D. Fraction of cells in each point of the cell cycle measured after 72h of treatment with 500nM JQ1 or DMSO (control). E. Cell death ELISA after 72h of treatment with 500nM JQ1 or DMSO (control). F. Scenescent cell fraction measured by ß-Gal activity after 72h of treatment with 500nM JQ1 or DMSO (control). G. BrdU ELISA performed after treatment of all medulloblastoma cell lines with 500nM JQ1 or DMSO (control) for 72 h.

To investigate the impact of JQ1 treatment on typical tumorigenic properties, we assessed viability, proliferation, apoptosis and senescence in the medulloblastoma cell lines, HD-MB3, Daoy, UW-228, ONS-76, D-283 and D-341, in culture. MTT assays were conducted after 48-120h of JQ1 treatment to assess cell viability. JQ1 treatment significantly reduced viabiltiy in medulloblastoma cells in culture (Fig. 2 A–C). Fifty percent inhibition of growth (IC50) in our cell line panel was observed at JQ1 concentrations between 78nM and >1μM (Fig. 2A). Lowest IC50 values occurred primarily after 72h of treatment. Interestingly, the *MYC*-amplified HD-MB3 cell line was among the most sensitive to JQ1 treatment. The Daoy and UW-228 cell lines were least sensitive to JQ1 with IC50 values well above 1μM. In comparison to the other cell lines, Daoy and UW-228, harbor *TP53* mutations. Previous studies using RNAi showed that the BET family member, BRD4, is required for transition through the cell cycle [16]. Consistent with this observation, cell cycle analysis in our medulloblastoma cell line panel revealed an increase in the percentage of cells in G1 after JQ1 exposure (Fig. 2D). These data suggest that BET bromodomain inhibition perturbs cells at the G1/S border. Treatment with JQ1 also increased the population of sub-G1 cells, suggesting that JQ1 induces apoptosis in a fraction of cells in the population (Fig. 2D). Analysis of treated cells using the cell death detection ELISA™ produced similar findings. The fraction of apoptotic cells was significant higher after JQ1 treatment in all cell lines except D-283 (Fig. 2E). JQ1 produced the strongest apoptotic response in the *MYC*-amplified cell lines, HD-MB3 and D-341. JQ1 treatment also enhanced cell senescence in most of the six medulloblastoma cell lines (Fig. 2F). Cell proliferation was analyzed using BrdU incorporation 72h after treatment, and showed significantly reduced proliferative capacity in most of the six medulloblastoma cell lines after JQ1 treatment (Fig. 2G). Taken together, the BET bromodomain inhibitor, JQ1, induces cell cycle arrest, senecence and apoptosis with a range of sensitivity across a panel of medulloblastoma cells.

### JQ1 treatment deregulates MYC and MYC target expression in medulloblastoma cell lines

**Figure 3:**
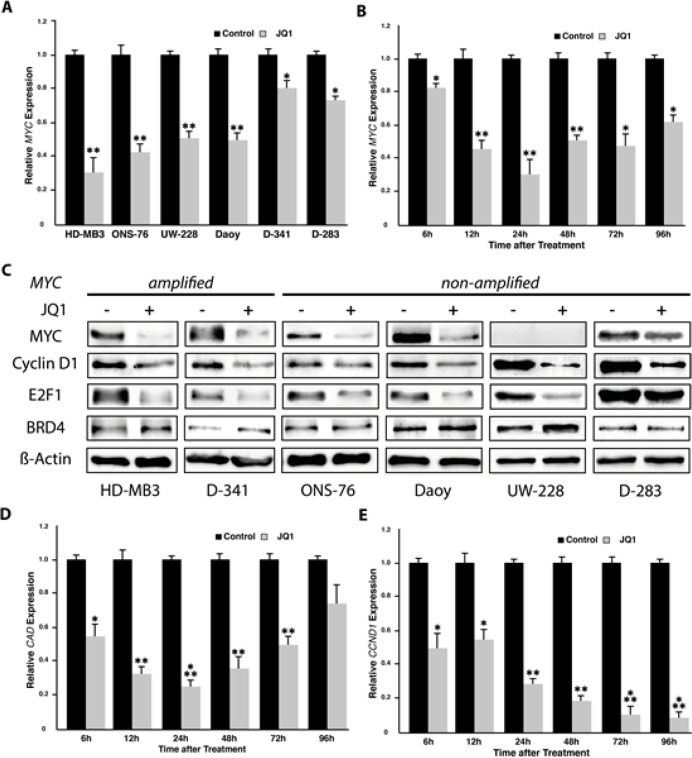
JQ1 treatment induces downregulation of MYC and its targets as well as of genes involved in cell cycle progression (cyclin D1 and E2F1) A. Analysis of *MYC* expression using real-time RT-PCR in all 6 medulloblastoma cell lines after 24h of treatment with 500nM JQ1. Analysis of *MYC* (B) and the MYC target gene, *CAD* (D), as well as *CCND1* (E) expression after a time course of 6-96h of JQ1 treatment (500 nM) using real-time RT-PCR. C. Expression of MYC as well as proteins selected from significantly affected genes in the expression array (cyclin D1 and E2F1, see Fig. 4) was assessed by western blotting of whole-cell lysates of medulloblastoma cell lines treated with 500nM JQ1 or DMSO control for 72h.

We next examined the effect of BET inhibitors on MYC functionality in several genetic backgrounds relevant for human primary medulloblastoma. We used the two *MYC*-amplified cell lines, HD-MB3 and D-341, along with three cell lines (ONS-76, Daoy, and D-283) lacking *MYC* amplifications and one cell line (UW-228) that chronically expresses low *MYC* levels to model three genetic backgrounds, and measured *MYC* and MYC target gene expression using real-time RT-PCR. JQ1 treatment reduced *MYC* transcript levels in all cell lines regardless of *MYC* status (Fig. 3A and 3B). *MYC* expression was reduced by over 80% in the *MYC*-amplified cell line, HD-MB3 (Fig. 3A and 3B). The reduction in *MYC* transcript levels in cell lines treated with BET inhibitor was also recapitulated at the protein for MYC (Fig. 3C). Since MYC is known to drive proliferative activity, transcriptional reprogramming via *MYC* suppression may significantly contribute to the growth inhibitory effects mediated by BET bromodomain inhibition. MYC is known to interact with the *CAD* promotor and increase expression of *CAD*, which encodes enzymes for pyrimidine nucleotide biosynthesis [19]. We assessed *MYC, CAD* expression using real-time RT-PCR in time course in HD-MB3 cells treated with JQ1 to evaluate the temporal relationship between *MYC* suppression and *CAD* downregulation in cell lines treated with JQ1. *MYC* expression declined from 6h to 24h, where it reached its low point, and then recovered slightly to an intermediate, but significantly reduced level (Fig. 3B). This slight recovery may be due to known autoregulatory feedback circuits of *MYC* [25]. *CAD* expression was also initially repressed, and mirrored the constant *MYC* decline from 6h to 24h, but then recovered almost to control level expression by 96h (Fig. 3D). Considering the observed defects in cell cycle progression at G1/S border we also analysed *CCND1* expression in time course. In contrast to *MYC* and *CAD, CCND1* was repressed with a slight delay, beginning at 12h, but was continuously repressed to approximately 10% of control cells by 72h, where it remained for the rest of the time course (Fig. 3E). These observations support MYC downregulation as a primary effector of growth inhibition by BET inhibition in MYC-driven tumor cells.

### JQ1 affects components of the p53 pathway and cell cycle control elements

**Figure 4:**
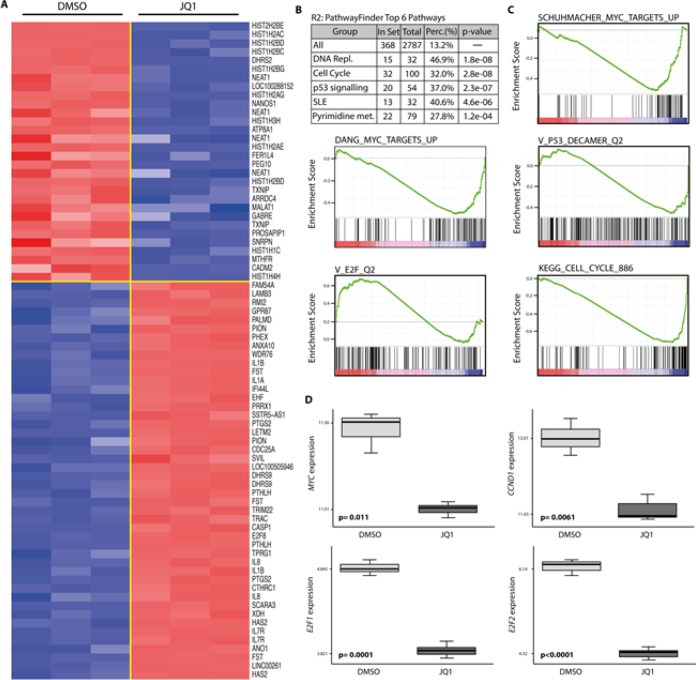
Treatment with JQ1 deregulates gene expression of MYC target genes as well as genes involved in cell cycle and p53 pathways A. Heatmap representation of the top 30 down- (blue) and up-regulated (red) genes (p < 0.001) in the HD-MB3 human medulloblastoma cell line following 24h treatment with 500nM JQ1. Data are presented in row-normalized form, with a range with ±3 standard deviations from median expression. B. The top 6 pathways deregulated by JQ1 treatment of HD-MB3 cells. C. Gene set enrichment analysis (GSEA) of JQ1-mediated expression changes in HD-MB3 cells, showing deregulation of a set of genes involving *MYC* (DANG_MYC_TARGETS_UP and SCHUMACHER_MYC_TARGETS_UP), *TP53* (V_p53_DECAMER_Q2), cell cycle (KEGG_CELL_CYCLE_886) and *E2F* (V_E2F_Q2). D. Expression differences in the *MYC, CCND1, E2F1* and *E2F2* genes in JQ1-treated and control (DMSO) cells are shown as box-plots.

**Figure 5:**
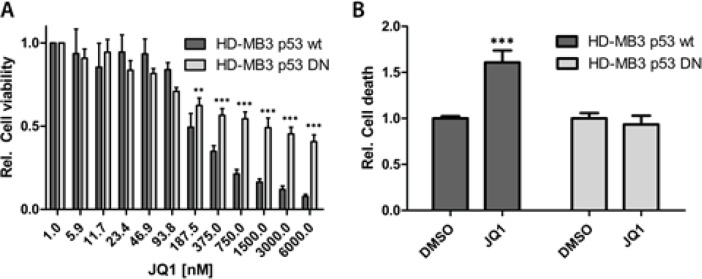
Enforced expression of a dominant-negative TP53 mutant abrogates the JQ1 effect A. HD-MB3 cells stably transfected with a dominant-negative TP53 mutant (HD-MB3-p53DN) or parental cell line with wt p53 were treated with the JQ1 concentrations indicated for 72h, then viability was assessed by MTT assay. B. The cell lines described in A were treated with either 500nM JQ1 or DMSO control for 72h, then the fraction of apoptotic cells was measured with the cell death ELISA.

Given the broad activity of JQ1 in medulloblastoma cells, we sought to identify potential mechanisms that could explain these phenotypic responses. We performed expression profiling of HD-MB3 cells lines treated with JQ1 or DMSO control medium using Affymetrix arrays. Cells were treated for 24h with 500nM JQ1 to identify early, and potentially direct, transcriptional targets of JQ1 treatment. Data were normalized, then the average differential expression score from the data was used to determine genes affected by JQ1 treatment. A total of 239 and 474 genes were up- and downregulated (*p-value* < 0.001 and pfp < 0.05), respectively, after JQ1 treatment. The top 50 differentially up- and downregulated genes are depicted in the heatmap (Fig. 4A). Using R2 Database (http://hgserver1.amc.nl/cgi-bin/r2/main.cgi), we compared most deregulated genes upon (+)-JQ1 treatment with a set of KEGG-Pathways to find pathways most significantly enriched with those genes. 368 out of 2787 pathways met our criteria of t-test p < 0.001. The top 3 pathways identified in this analysis were DNA replication, cell cycle and the p53 pathway, with a 32-47% overlap and p<2.8e-08 (Fig. 4B). Next we used GSEA to assess the effects of JQ1 on transcriptional programs regulated by MYC, the E2F family, cell cycle and TP53. We interrogated our data for the statistically significant enrichment of published, validated gene signatures. The majority of gene sets associated with MYC, cell cycle, the E2F family and TP53 were statistically enriched among the genes that were downregulated by BET inhibition in HD-MB3 cells (Fig. 4C). As medulloblastoma cell lines harboring *TP53* mutations were less responsive towards JQ1 treatment than those with wildtype *TP53*, we further investigated the functional implication of *TP53* status for JQ1 efficacy. We generated the HD-MB3-p53DN cell line, and treated these with JQ1. Sensitivity to JQ1 was diminished by enforced expression of the dominant-negative TP53 (Fig. 5A). Furthermore, enforced expression of the dominant-negative TP53 mutant completely abolished the ability of JQ1 (500nM, 72h) to induce apoptosis (Fig. 5B). These findings further support the functional importance of *TP53* status for JQ1 efficacy in medulloblatoma cells.

A number of key G1-associated cell cycle genes were deregulated in HDMB-3 cells after 24h of JQ1 treatment. This result is consistent with our observation of defective cell cycle progression in JQ1-treated medulloblastoma cell lines (Fig. 2D) as well as the dramatic supression of *CCND1* expression in HD-MB3 cells treated in time course (Fig. 3E). Taken together, our data support that JQ1 treatment deregulates the *MYC* program as well as the cell cycle and p53 pathways in medullloblastoma cells.

### BRD4 knockdown phenocopies the effects of JQ1 treatment in medulloblastoma cells

**Figure 6:**
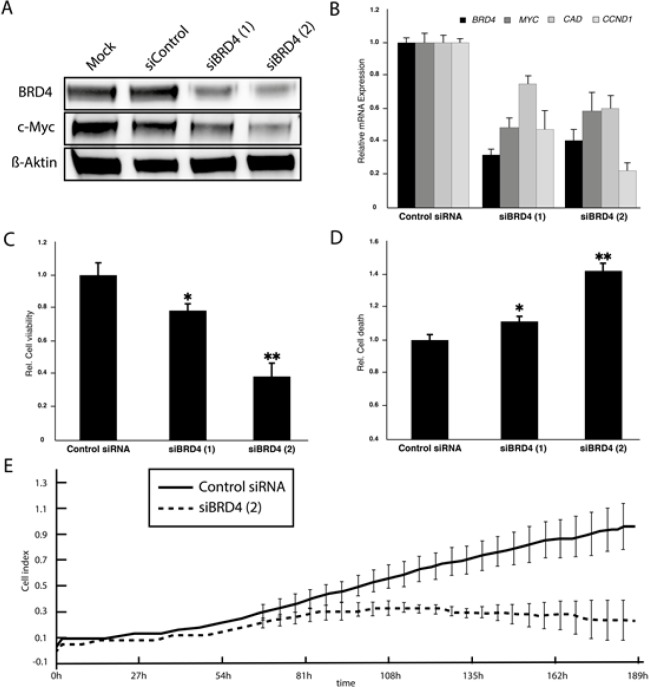
siRNA-mediated BRD4 knockdown phenocopies JQ1 effects in HD-MB3 cells **A.** Protein expression in western blots is shown for HD-MB3 cells, which harbor a *MYC* amplification, 48h after either mock transfection (mock) or transfection with a control siRNA or an siRNA specific for BRD4 (siBRD4). B. Real-time RT-PCR of *BRD4* expression in HD-MB3 cells 48h after transfection with a control siRNA or an siRNA specific for BRD4 (siBRD4). C. Proliferation of cells monitored for 8 days in real time using the Xcilligance system after siRNA-mediated BRD4 knockdown compared with cells transfected with control siRNA.

Previous studies have focused on the role of the specific BET family member, BRD4, which is most selectively inhibited by JQ1. Moreover, a recent siRNA screen to identify synthetic lethal interactions with *MYC* overexpression revealed *BRD4* as a candidate gene [26]. Therefore, we transiently transfected the HD-MB3 cell line with two siRNAs directed against BRD4 or with transfection control alone and examined the effect of BRD4 knockdown on the same tumorigenic parameters we assessed after JQ1 treatment. A significant knockdown of BRD4 was achieved on both the mRNA and protein levels 72h after transfection (Fig. 6A+B). As we observed following JQ1 treatment, BRD4 knockdown resulted in a downregulation of MYC mRNA and protein expression in real-time RT-PCR and western blots, respectively (Fig. 6A+B). Cell viability of HD-MB3 was significantly reduced and the fraction of apoptotic cells was elevated after transfection with both siRNAs (Fig. 6C+D). The siRNA siBRD4(2) more effectively knocked down BRD4 expression on protein level than siBRD4(1). We, therefore, decided to monitor cells transfected with siBRD4(2) or control siRNA for 190h using the Xcelligance system. HD-MB3 cells transfected with siBRD4(2) proliferated more slowly than cells transfected with control siRNA (Fig. 6C). Taken together, BRD4 knockdown reduced both MYC expression and viability in the group 3-derived medulloblastoma cell line, HD-MB3, and therefore, phenocopies the effects of JQ1 treatment.

### JQ1 has antitumoral activity against human medulloblastoma xenografts in mice

**Figure 7:**
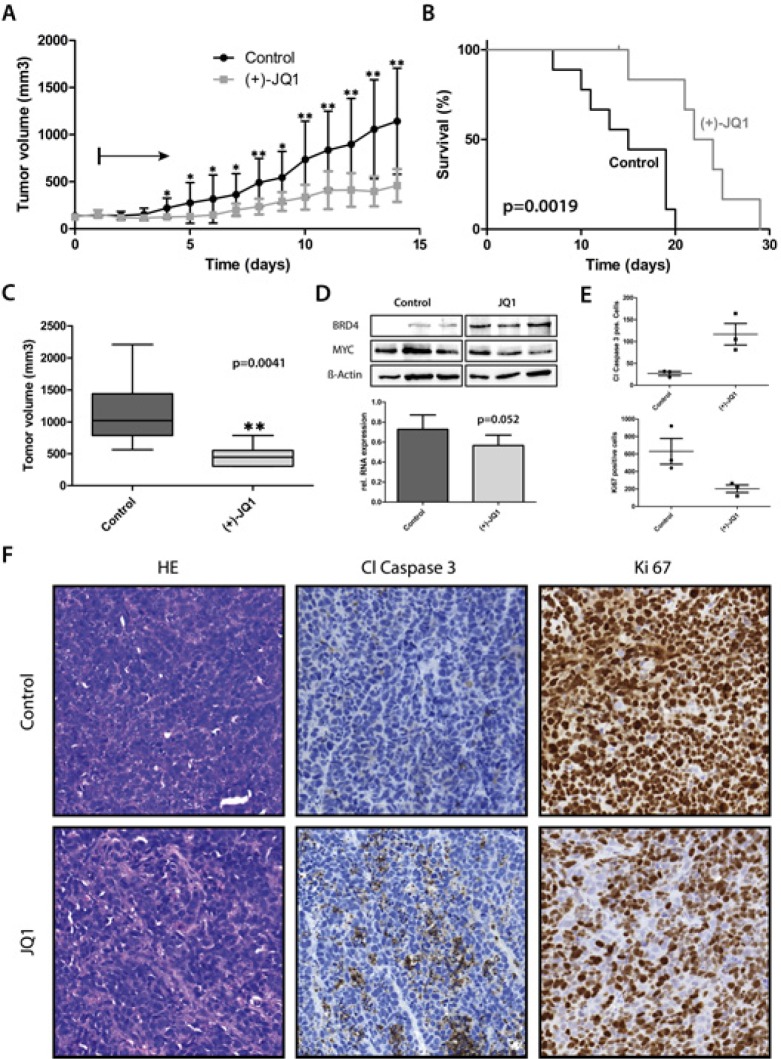
JQ1 treatment significantly prolonged survival and decreased tumor volume in mouse medulloblastoma xenografts A. Tumor volume of subcutaneous xenografts *of MYC*-amplified HD-MB3 cells in nu/nu mice treated with JQ1 (50mg/kg body weight per day) or control (DMSO). Black arrow indicates beginning of JQ1 treatment. B. Kaplan-Meier analysis of JQ1-treated and control mouse cohorts. C. Box-plots show tumor volume after 14 days of treatment. D. Western blots show MYC and BRD4 expression in xenograft tumors, with β-actin used as a loading control. Protein expression is shown for all 3 tumors each from mice treated with JQ1 (50mg/kg BW) or DMSO for 2 days twice dayly. Bar graph shows mean (± S.D.) *MYC* expression measured in the 3 tumors by RT-PCR. E+F. Plots represent quantified expression of cleaved caspase 3 to identify apoptotic cells (p<0.05) or Ki67 to identify proliferating cells (p<0.05) from immunohistochemically stained xenograft tumors treated as described in D with JQ1 or DMSO. Selected micrographs show representative immunostaining in addition to hematoxylin & eosin staining (HE) for all 6 xenograft tumors.

To further investigate the antitumor effects of BET inhibition *in vivo*, we explored a xenograft model of high-risk medulloblastoma. We used the most recently established group 3 cell line, HD-MB3, to recapitulate the tumor biology of this subgroup as closely as possible. HD-MB3 cells were injected subcutaneously into the flanks of immunocompromised mice. After palpable xenograft tumors were established, JQ1 was administered intraperitoneally to 7 mice once a day at 50 mg/kg body weight for a total of 28 days or until tumor size exceeded 1000mm^3^. A cohort of 7 mice harboring xenograft tumors were injected with vehicle alone in the same injection regimen. Tumors grew significantly slower in JQ1-treated mice compared with vehicle-treated controls (Fig. 7A). Kaplan-Meier analysis showed that tumor growth to an average of 1,000 mm3 was delayed by 8 days in the treatment group compared with the vehicle control group (Fig. 7B). Thus, JQ1 treatment led to a highly significant increase in survival in the treatment group (Fig. 7B). One of 7 mice in the treatment group was euthanized because of a bleeding tumor at day 15 of treatment and was, therefore, omitted from Kaplan-Meier analysis. The average tumor volume was significantly smaller in the JQ1-treated group from day 4 onward to day 14 (Fig. 7C). Intensified JQ1 treatment consistently downregulated MYC expression at both mRNA and protein levels in HD-MB3 tumors (Fig. 7D). Immunohistological examination of xenograft tumors showed that JQ1 increased the number of apoptotic cells and reduced the number of proliferating cells in tumors (7E and 7F). These data demonstrate that BET inhibition results in significant *in vivo* antitumoral activity in high-risk MYC-driven medulloblastoma.

## DISCUSSION

Medulloblastoma is the most common brain tumor in children and arises from primitive neural cells of the cerebellum [1]. The majority of children with high-risk disease succumb to medulloblastoma despite intensive chemotherapy, surgery and radiation [2]. Moreover, surviving patients often suffer from long-term sequelae due to the aggressive treatment regimens. Targeted treatment approaches are, thus, clearly needed for these patients. The important role of the *MYC* oncogene in high-risk medulloblastoma tumorigenesis makes it a compelling, though until recently undruggable, target for drug development. Here we show that selectively targeting *MYC* using JQ1, a small molecule inhibiting BET bromodomains, displayed broad antineoplastic effects in medulloblastoma *in vitro* and *in vivo*.

It was reported that siRNA knockdown of BRD4, the main target of JQ1, reduces expression of key G1- and growth-associated genes, leading to cell cycle arrest in G1 and apoptosis in HeLa cells [16]. The same effect was observed in previous studies examining the effect of BET inhibition in different cancer entities [20, 21, 27, 28]. Here we show that targeting BRD4 either pharmaceutically with JQ1 or by siRNA knockdown was effective at reducing the viability of medulloblastoma cell lines. As has been described before in cells derived from other tumor entities, we showed here that JQ1 induced medulloblastoma cells to accumulate in the G1 phase of the cell cycle. The highest incidence of apoptosis occurred in cells harboring *MYC* amplifications, namely HD-MB3 and D-341, indicating the importance of *MYC* status for JQ1 efficacy [29]. Interestingly, in cell lines in which JQ1 did not induce apoptosis, induction of senescence was observed. Cell lines differed considerably in respect to their sensitivity to JQ1. Until now, the capability and capacity to respond to JQ1 has primarily been attributed to the extent of endogenous *MYC* expression [28]. In glioblastoma though, Cheng et al (2013) postulated that p21 provided partial protection against JQ1 when genetically manipulated [27]. Interestingly, one cell line (Daoy) with only a minor response to JQ1 treatment in our study, expressed lower levels of BRD4 than the other cell lines. Inconsistant with the assumption that this lower BRD4 expression may be causal for the reduced sensitivity, Daoy exhibited the same level of *MYC* and MYC downregulation after JQ1 treatment as cell lines which responded well to JQ1. The other cell line (UW-228) with a minor response to JQ1, did not express detectable MYC levels in western blots. Thus, poor JQ1 response in UW-228 can probably be attributed to the absence, at this level of detection, of the primary drug target from the cellular background. Interestingly, both the UW-228 and Daoy cell lines harbor *TP53* mutations. Combining these results with our expression profiling findings, we hypothesize that the mutant *TP53* could impede response to JQ1. To substantiate p53 dependance of JQ1 effects, we showed that enforced expression of a dominant-negative *TP53* mutant in a cell line formerly sensitive to JQ1 treatment impaired the JQ1 effect. These results are concordant with observations recently published by Shwu-Yuan Wu et al., who described important interactions between BRD4 and TP53 [11]. They showed that BRD4 bound to and recruited TP53 to promoters of genes known to be regulated by TP53. Fewer than 10% of sporadic medulloblastomas harbor *TP53* mutations [30]. However, *MDM2* amplification or overexpression, which increases TP53 degradation, is frequently observed in medulloblastomas with wildtype *TP53* [31]. Based on these observations and previous work where we demonstrated that pharmacologically inhibiting MDM2 in medulloblastoma cells with wildtype *TP53* reactivated p53 activity, it may be advantageous to investigate possible synergies between JQ1 and MDM2 inhibitors, such as nutlin-3 [32]. As *TP53* mutations are among the most recurrent alterations in human cancer entities, additional evidence of the dependence of the activity of BET inhibition on *TP53* status should be further investigated in other tumor entities as well.

Looking at previous studies, BET inhibition appears to be highly selective and affect only a few genes. One compelling feature of JQ1 is the ability to effectively downregulate *MYC* [20], which is a gene long thought to be difficult if not impossible to target. JQ1 was highly effective against preclinical models of several types of cancers with recurrent *MYC* amplifications, including acute myeloid leukemia, Burkitt's lymphoma and multiple myeloma, and efficacy was directly related to the extent of MYC suppression by treatment [20, 21]. Supporting these previous findings, medulloblastoma cells exhibited a similar reduction of *MYC* expression following BET inhibition here, and the strongest apoptotic induction occurred in *MYC*-amplified cell lines. We also observed downstream effects of *MYC* suppression. *CAD*, a gene known to be regulated by MYC and BRD4, expression was similarly downregulated after JQ1 treatment. This may have resulted from a direct inhibitory effect of JQ1 on the interaction of BRD4 with the *CAD* promoter in addition to the secondary effect of *MYC* downregulation. Taken together, BET inhibition via JQ1 downregulates MYC as well as MYC targets in medulloblastoma cells.

To gain further functional insight into the working mechanisms of BET bromodomains, we performed expression profiling of medulloblastoma cells with and without JQ1 treatment. Gene set enrichment analysis using KEGG pathways revealed that targets affected by BET inhibition were involved in cell cycle progression, the p53 pathway and DNA replication. This fits our observation that JQ1 appears to disrupt the cell cycle by inducing G1 arrest and apoptosis. We also observed that expression of two key factors in G1 progression, cyclin D1 and E2F1, were repressed, mirroring the reduction in MYC expression, by JQ1 treatment. Since siRNA-mediated depletion of the BRD2 BET protein has been described to reduce transcription of cyclin D1 in human cells [14], we investigated the result of siRNA-mediated BRD4 knockdown on *CCND1* regulation in medulloblastoma cells. BRD4 knockdown reduced *CCND1* expression similarly to JQ1 treatment. Although investigation into the mechanisms by which BRD4 influences cyclin D1 expression was beyond the scope of this paper, further investigation of this interaction could be of clinical relevance, considering the importence of the cyclin D family in tumor maintenance [33]. Our findings suggest that not only *MYC*, but a variety of genes involved with the p53 pathway and in cell cycle control are affected by JQ1 treatment. These results are concordant with previously published results for JQ1 action on neuroblastoma cells, which described supression of a variety of genes including the E2F family [28]. These new insights implicate the BET protein family in the fine control of cell cycle regulation and progression, emphasizing the benefit of further functional investigation of this family for tumorigenic control mechanisms.

With the aim to generate preclinical evidence for JQ1 efficacy in an animal model for high-risk medulloblastoma, we treated subcutaneous xenografts of cells derived from a group 3 medulloblastoma in nude mice with JQ1. Given that JQ1 has no difficulty passing the blood-brain barrier, JQ1 should have similar effects on tumors regardless of their localization, and not present a problem for reaching medulloblastoma in the brain. However, testing in orthotopic medulloblastoma models could provide additional preclinical information prior to entry into clinical trials. The HD-MB3 cell line is well-described and one of the few medulloblastoma cell lines originating from a *MYC*-amplified group 3 tumor [34]. We only used this cell line, since this model is recently established, and is most probably the closest recapitulation of Group 3 medulloblastoma biology when compared to more established cell lines [34]. JQ1 potently inhibited xenograft tumor growth, using these cells, and significantly prolonged mouse survival. Similarly to our *in vitro* findings, JQ1 reduced *MYC* expression in xenograft tumors. Interestingly, JQ1 resulted in elevated BRD4 protein expression in treated tumors. This observation may indicate that an endogenous feed-back loop could be initiated by BRD4 inhibition at least in some cellular backgrounds. To avoid such feedback mechanisms, further investigations about optimal dosing schedules for BET inhibitors might be conducted. Our results give conclusive evidence that BET inhibition via JQ1 is effective in reducing medulloblastoma growth in a preclinical mouse model for high-risk medulloblastoma.

Future clinical testing of BET inhibitors in high-risk medulloblastoma is clearly necessary to provide the ultimate proof of the usefulness of this therapeutic strategy for the treatment of this highly aggressive pediatric malignancy. Investigating possible synergies between BET inhibitors and other therapeutic agents in clinical use for medulloblastoma treatment should, therefore, be addressed in future studies. As mentioned above, combining BET inhibition with targeted therapies restoring TP53 expression, such as MDM2 inhibitors, could provide one possibly synergistic option. Many therapeutic strategies now used to treat medulloblastoma, such as radiation, induce DNA damage in tumor cells. DNA damage normally activates a signalling network that blocks cell cycle progression, recruits DNA repair factors or triggers senescence or apoptosis [35]. It is well established that this DNA damage response is abrogated in many malignancies in such a way that tumor cells manage to maintain their proliferative capacity [36]. BRD4 was recently shown to insulate chromatin from DNA damage signalling [37], indicating that combining DNA-damaging agents with BET inhibitors, such as JQ1, may increase DNA damage response in medulloblastoma cells. Exploring such putative synergies in clinical and preclinical investigations should fine-tune optimal conditions for JQ1 administration.

Intensive research in the past years has revealed BET bromodomain inhibition as a potent course of action to inhibit *MYC* and induce antitumoral effects in different malignancies [20-22, 27, 28]. Our observations explored the feasibility of treating high-risk medulloblastoma patients with JQ1 in preclinical models. The survival advantage observed in mouse xenograft models of high-risk medulloblstoma suggests that JQ1 has the potential to generate a measurable response in this patient subgroup, which have dismal prognoses with current treatment regimens, and thus, should be considered for entry into clinical testing.

## MATERIAL AND METHODS

### JQ1 treatment of cell lines in culture

The HD-MB3, ONS-76, DAOY and UW-228 human medulloblastoma cell lines were grown in RPMI 1640 supplemented with 10% FCS, L-glutamine, and antibiotics [38]. Medium for HD-MB3 was also supplemented with 1% nonessential amino acid solution. The human medulloblastoma cell lines, D-341 and D-283, were cultured in Eagle's Minimum Essential Medium supplemented with 10% FCS and antibiotics. The identity of all cell lines was verified by STR genotyping performed by the German Collection of Microorganisms and Cell Cultures (DSMZ, Braunschweig, Germany). HD-MB3-p53DN cells were generated by transfecting HD-MB3 with pMSCV-puro-p53DD (Addgene, #K1062-1, Cambridge, MA, USA), and selected for stable transfectants with 2 μg puromycin/ mL full medium. Pure (+)-JQ1 sterioisomer (BPS Bioscience, San Diego, CA, USA), which is referred to as JQ1 throughout this paper, was aliquoted for single use and stored at −20°C as stock solution (10 mM in DMSO). Cells were exposed to 0–6 μM JQ1, prepared as serial dilutions in full medium, for the periods indicated. The final dimethyl sulfoxide (DMSO) concentration was kept at or below 0.2%.

### Real-time reverse transcriptase-PCR

Total RNA was isolated from cells using the RNeasyMini kit (Qiagen, Hilden, Germany), and cDNA synthesis was performed using the SuperScript reverse transcription kit (Invitrogen, Darmstadt, Germany). *CAD, CCDN1* and *MYC* expression were monitored using Assays-on-Demand™ (Applied Biosystems, Foster city, CA, USA). Expression values were normalized to the geometric mean of *GAPDH* [39]. Data analysis and error propagation were performed using the qbase^PLUS^ software version 1.5 (http://www.biogazelle.com).

### Western blot analysis

Protein lysates were extracted from cells, separated on SDS-PAGE and electrotransferred as described in Cimmino et al. [40]. Membranes were blocked 1h with 5% nonfat dry milk in TBS-T, then incubated with primary antibodies in 5% nonfat dry milk in TBS-T overnight or up to 24h at 4°C. Primary antibodies against the following proteins were used: MYC (1:1000, #9402, Cell Signaling, Danvers, MA, USA), BRD4 (1:200, H-250, sc-48772, Santa Cruz, Dallas, TX, USA), Cyclin D1 (1:200, H-295, sc-753, Santa Cruz), E2F1 (1:200, AF4825, R&D Systems, Minneapolis, MI, USA) and B-actin (1:2000, Sigma-Aldrich, St. Louis, MO, USA) as the loading control. After washing twice with TBS-T, membranes were incubated 1h at room temperature with secondary antibodies diluted 1:2000 in 5% nonfat dry milk in TBS-T. Horseradish peroxidase (HRP)-conjugated anti-rabbit IgG (GE Healthcare, Fairfield, USA) or HRP-conjugated anti-mouse IgG (GE Healthcare) were used as secondary antibodies. Proteins were visualized using the ECLplus western blotting detection kit (Amersham, Amersham, UK) and analyzed on a FusionFX7 detection device (Vilber Lourmat, Eberhartzell, Germany).

### Cell proliferation, death, viability, senencence and Cell cycle analysis

Medulloblastoma cell lines were seeded onto 96-well plates (2 × 10^3^ per well) in triplicate for all assays, and incubated for 24h to permit surface adherence. Viability was assessed in time course after treatment with 1nM-6μM JQ1 for 24, 48, 72 and 96h using the 3-(4,5-dimethylthiazol-2-yl)-2,5-diphenyltetrazolium bromide (MTT) assay (Roche, Basel, Switzerland), according to the manufacturer's protocol. The IC50 was calculated using GraphPad Prism 5.0 (GraphPad Software Inc., San Diego, CA, USA). Apoptosis, proliferation and senescence were assessed after identical time-course treatment with 500 nM JQ1 using the Cell Death and BrdU ELISAs (Roche) and the fluorometric SA-ß-gal activity assay (Cell Biolabs, San Diego, CA, USA), respectively. All assays were performed according to the manufacturer's protocols. For cell cycle analysis, cell lines were cultured 48h with 500 nM JQ1 or DMSO control in 35mm plates at 5 × 10^7^ cells/plates. Cells were removed by trypsinization and washed 3 times with PBS, then incubated with propidium iodide for 15 min to stain DNA. Cellular DNA content was analyzed in an FC500 flow cytometer (Beckman Coulter). All experiments were independently performed at least 3 times, if not otherwise indicated.

### BRD4 knockdown and Xcelligence viability assay

To assess cellular survival after BRD4 knockdown, we transfected HD-MB3 cells with siRNA against BRD4. HD-MB3 cells were seeded at 5 × 10^4^ on a 6-well plate and incubated for 24h. HD-MB3 cells were transiently transfected with either an siRNA directed against BRD4 (siBRD4 (1), Cat. No. s23901, and siBRD4 (2), Cat. No. s23902, Ambion, Austin, TX, USA) or the scrambled control siRNA at a final concentration of 20 nmol using the Hiperfect transfection reagent (Qiagen, Hilden, Germany) according to the manufacturer's protocol. Mock control using only transfection reagent was also performed. Protein and mRNA expression were analyzed using western blotting and real-time RT-PCR, respectively, as described above. Cell viability and cell death were analyzed after 72h of transfection by MTT assay and cell death ELISA as described above. Since siBRD4 (2) showed the most effective BRD4 knockdown, we assessed cellular survival after transfection with this siRNA in real time using the Xcelligence system (Roche). HD-MB3 cells were plated in triplicate at 2 × 10^3^ cells/well onto 96-well Xcelligence microelectronic cell sensor plates, and cultured overnight in antibiotic-free complete media. Cells were transiently transfected with 20 nmol of either siBRD4 or control siRNA or mock transfected, then adherence to the culture plates was continuously monitored for 190h to assess cellular survival.

### Tissue microarrays and immunohistochemistry

Tissue microarrays were prepared from paraffin-embedded tissue specimens from 115 primary medulloblastomas and 14 cerebellum samples [32]. For the array, 3 different tissue cores were taken from each formalin-fixed, paraffin-embedded tumor block using a manual device (Beecher Instruments, Sun Prairie, WI, USA), and 2μm sections were cut from the microarray for immunohistochemical analyses. Sections from 2 additional formalin-fixed paraffin-embedded human primary medulloblastoma samples (2μm) and the formalin-fixed, paraffin-embedded xenograft tumors (5μm) from JQ1-treated and untreated nude mice were also analyzed immunohistochemically. Immunohistochemical staining was conducted as previously described [41]. In brief, formalin-fixed paraffin-embedded tissue sections were deparaffinized by routine techniques, and placed in 200 μL of target retrieval solution (pH, 6.0; Envision Plus Detection Kit; Dako, Glostrup, Denmark) for 20 min at 100°C. After cooling for 20 min, slides were quenched with 3% H2O2 for 5min before incubating with primary antibody using a Dako Autostainer (Dako Cytomation). Primary antibodies against BRD4 (1:200, sc-48772, Santa Cruz), cleaved caspase 3 (1:200, Cell Signaling) and Ki67 (1:25, DAKO) were used. BRD4 expression was evaluated by 2 independent researchers using a semiquantitative scoring system on microarrays. In brief, the number and intensity of positive cells were counted and scored between 0 and 3 (0, no positive nuclei; 1, 1% – 20% nuclei display intense staining or more nuclei display weak staining; 2, 20% – 80% intense staining or more nuclei display moderate staining; 3, 80% – 100% nuclei display intense staining). Samples with scores of 1 – 3 were considered as expressing the target protein.

### Affymetrix microarray analysis

HD-MB3 cells plated at 5 × 10^4^ cells/well in 6-well plates, then after 12h for attachment, were treated in triplicate with medium containing 0.2% DMSO (control) or 500nM JQ1 for 24h. Total RNA was extracted using the RNeasyMini kit (Qiagen, Hilden, Germany), and used to confirm at least a 50% decrease in *MYC* expression in the JQ1-treated cells using real-time RT-PCR. Three replicas were treated and analyzed. Samples were profiled using the genechip Affymetrix Human Gene Expression Array (HG-U133 Plus 2.0, Affymetrix, Santa Clara, CA, USA) at the Centre for Medical Biotechnology at the University Hospital Essen using established protocols. Microarray. CEL files were normalized and summarized to gene levels to conduct gcRMA normalization using the Bioconductor repository of statistical tools [42]. Probes for which the log2 expression was < 4 in at least 5 of the 6 samples, were considered underexpressed and filtered out of the dataset. Expression data from a total of 23376 probes remained for analysis after filtering. Differential expression analysis was performed using the Rank Product analysis package v 2.13 in R [43]. Hierarchical clustering was performed on the Manhattan distance of log2 expression values for either all or the 100 most differentially expressed genes to visualize differential gene expression in HD-MB3 cells following JQ1 treatment. Gene set enrichment analysis was performed using the GSEA v2.0 software from www.broadinstitute.org/gsea [44]. Genes were ranked using a signal:noise ranking metric (the difference of the means of the populations compared scaled by the standard deviation). The c2.cp.kegg.v3.1.symbols.gmt, c2.all.v3.1.symbols.gmt and c5.all.v3.1.symbols.gmt (www.broadinstitute.org/gsea) gene sets were used in this study.

### Statistical analysis

We used the R2 visualization and analysis platform (http://hgserver1.amc.nl/cgi-bin/r2/main.cgi) to re-analyze expression data from human primary medulloblastoma microarrays (Künkele et al. 2012) and analyze expression data from JQ1-treated and control HD-MB3 cells. SPSS, version 18.0 (IBM SPSS) was used for further statistical analysis. The student's two-sided t-test was used to compare all interval variables, and the chi-square test was used for the comparison for all categorical variables. Graph Pad Prism 5.0 (GraphPad Software Inc.) was used to perform Kaplan-Meier survival analysis with log-rank statistics on treated and untreated mouse cohorts.

### JQ1 treatment of xenograft tumors in nude mice

HD-MB3 medulloblastoma cells were cultured to 80% confluency, harvested and suspended in 200 μL Matrigel™ (BD Bioscience, Heidelberg, Germany) for subcutaneous inoculation (2 × 10^7^ cells per mouse, n=14 mice) into the left flank of 4-week-old female athymic (nu/nu) mice. Mice were randomly assigned to either JQ1 or vehicle control groups (n =7 mice per group) after tumors reached 150 – 200 mm^3^ in size. We used a JQ1 treatment regimen previously been shown to be safe for control mice and effective against different malignancies in mouse models [21]. JQ1 or vehicle control was administered daily by intraperitoneal injection at a dose of 50mg/kg body weight. Tumor growth was monitored using a caliper and tumor volume was calculated using the formula (breadth × length × height)/2. Mice were euthanized by cervical dislocation when tumor size exceeded 1000mm^3^. In mice, whose xenograft tumors were to be examined for *MYC* downregulation, 4 doses of 50 mg JQ1 per kg body weight were administered at 0, 12, 24 and 36 h over a 2-day course. Mice were euthanized by cervical dislocation 4 h after the last JQ1 dose. Xenograft tumors were excised from all mouse treatment cohorts, and divided into halves. Half the tissue was snap-frozen in liquid nitrogen then stored at −80°C and the other half was formalin-fixed and paraffin-embedded. All animal experiments were performed in accordance with the Council of Europe guidelines for accommodation and care of laboratory animals, and protocols were approved by the Ethical Commission for Animal Experimentation at the University Hospital Essen.

## Supplementary Figures


