# Supplementary material for: BET bromodomain protein inhibition is a therapeutic option for medulloblastoma
Source: Oncotarget. 2013 Oct 27;4(11):2080–95. doi: 10.18632/oncotarget.1534 (PMC3875771; doi:10.18632/oncotarget.1534)
Supplement: Supplementary file 1 [file oncotarget-04-2080-s001.pdf]

SUPPLEMENTARY FIGURES

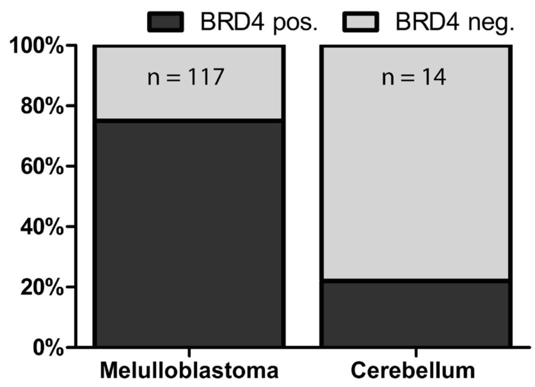

Supplementary Figure 1: BRD4 is strongly expressed in primary medulloblastomas. Tissue microarrays were prepared from paraffin-embedded tissue specimens from 115 primary medulloblastomas and 14 cerebellar samples. High-level BRD4 expression was observed in 75% of the medulloblastomas and only 22% of cerebellar samples analyzed.

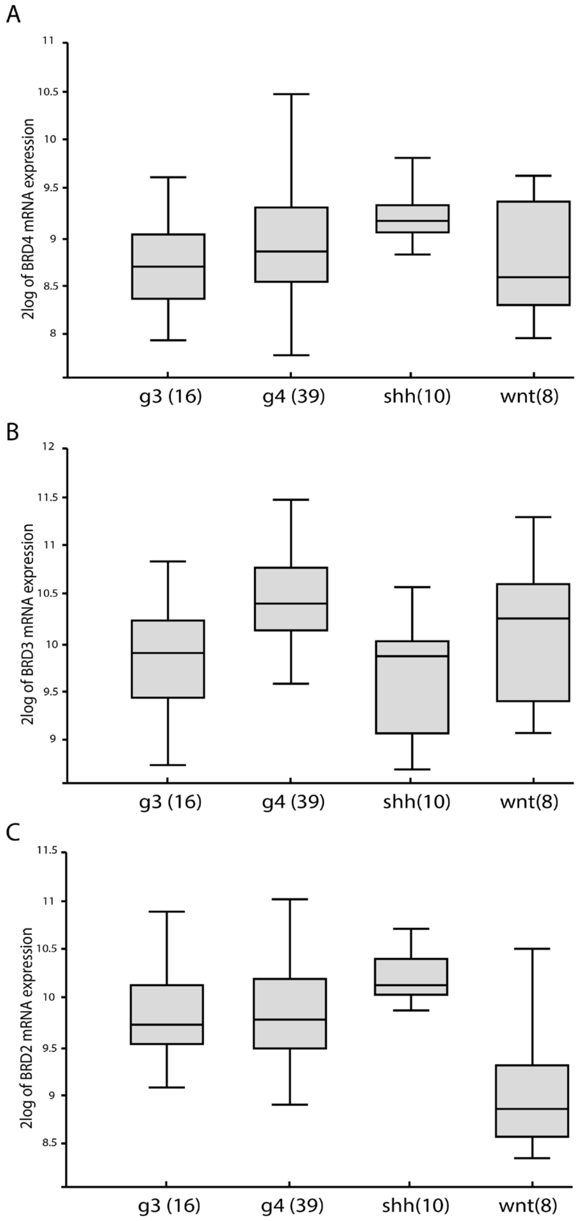

Supplementary Figure 2: All 4 medulloblastoma subgroups express high levels of BRD 2, 3 and 4. Robinson, et al. previously published Affymetrix global mRNA expression profiles from 76 primary medulloblastomas (Robinson G et al. Novel mutations target distinct subgroups of medulloblastoma. Nature. 2012; 488(7409):43-48.). We reanalyzed the data in a subgroup-specific manner (A,BRD4; B,BRD3; C, BRD2). Expression levels of BRD2 and BRD4 in Shh medulloblastomas were slightly higher (A and C) than the other three subgroups. BRD3 expression was highest in Group 4 medulloblastomas (B).
